# Flow-mediated plasticity in the expression of stickleback nesting glue genes

**DOI:** 10.1002/ece3.1016

**Published:** 2014-03-13

**Authors:** Paul J Seear, Megan L Head, Ceinwen A Tilley, Ezio Rosato, Iain Barber

**Affiliations:** 1 Department of Biology, College of Medicine, Biological Sciences and Psychology, University of LeicesterLeicester, LE1 7RH, U.K; 2 Division of Evolution, Ecology and Genetics, Research School of Biology, Australian National UniversityCanberra 0200, Australia; 3 Department of Genetics, College of Medicine, Biological Sciences and Psychology, University of LeicesterLeicester, LE1 7RH, U.K

**Keywords:** Adaptation, animal construction, glue, nest-building behavior, phenotypic plasticity, spiggin, sticklebacks

## Abstract

Nest construction is an essential component of the reproductive behavior of many species, and attributes of nests – including their location and structure – have implications for both their functional capacity as incubators for developing offspring, and their attractiveness to potential mates. To maximize reproductive success, nests must therefore be suited to local environmental conditions. Male three-spined sticklebacks (*Gasterosteus aculeatus*) build nests from collected materials and use an endogenous, glue-like multimeric protein – “spiggin” – as an adhesive. Spiggin is encoded by a multigene family, and differential expression of spiggin genes potentially allows plasticity in nest construction in response to variable environments. Here, we show that the expression of spiggin genes is affected significantly by both the flow regime experienced by a fish and its nesting status. Further, we show the effects of flow on expression patterns are gene-specific. Nest-building fish exhibited consistently higher expression levels of the three genes under investigation (*Spg-a*,*Spg-1,* and *Spg-*2) than non-nesting controls, irrespective of rearing flow treatment. Fish reared under flowing-water conditions showed significantly increased levels of spiggin gene expression compared to those reared in still water, but this effect was far stronger for *Spg-a* than for *Spg-1* or *Spg-2*. The strong effect of flowing water on *Spg-a* expression, even among non-nesters, suggests that the increased production of spiggin – or of spiggin rich in the component contributed by *Spg-a* – may allow more rapid and/or effective nest construction under challenging high flow conditions.

## Introduction

The construction of a suitable nest is an essential component of the reproductive behavior of many animals (Collias and Collias 1984; Hansell 2000, 2005). A major function of nests is to provide protection against adverse environments, so animals are expected to construct nests that are suited to prevailing local conditions; for example, the incorporation of insulation material into bird nests covaries with local thermal regimes (McGowan et al. 2004; Rohwer and Law 2010; Mainwaring et al. 2012), and both nest-building fish and birds orientate the entrances of constructed nests in relation to prevailing dynamic currents (Yuan 1996; Vinyoles et al. 2002). Patterns of nesting behavior that are suited to local environments could therefore reflect evolutionary adaptations, or they may arise through phenotypic plasticity in response to prevailing local conditions (Refsnider and Janzen 2012; Heenan 2013). For example, common gobies (*Pomatoschistus microps*) manipulate the size of the nest entrance hole and the amount of covering substrate in response to local dissolved oxygen conditions and the presence of predators (Jones and Reynolds 1999a,b1999b), and blue tits (*Cyanistes caeruleus*) adjust the insulatory capacity of nests depending on ambient temperatures experienced during the building phase (Britt and Deeming 2011; Deeming et al. 2012). Such plasticity in nesting behavior might be expected to be particularly beneficial for animals that construct nests in temporally variable or unpredictable environments, as it may allow them to exploit a wider range of nesting opportunities, or to rapidly adjust nest structure in response to changing ecological conditions (Barber 2013).

For animals building nests in aquatic ecosystems, water flow can impose a major selective force, and aquatic organisms are often adapted to prevailing local flow conditions (Lytle and Poff 2004; Haas et al. 2010). Water flow rates in fluvial environments, however, can be highly variable over a range of timescales, and organisms inhabiting such ecosystems often exhibit patterns of behavioral plasticity to maximize success (Bennett et al. 2002). In addition to this natural variation, flow regimes are also increasingly subjected to anthropogenic activities such as dam construction, water abstraction, and channel modification (Pringle 2001), as well as by changes in temporal patterns of rainfall associated with climate change (Döll and Zhang 2010). All of these factors potentially interfere with critically important behaviors, including feeding, reproduction, and migration, which ultimately impact population sizes and community structure (Bunn and Arthington 2002; Poff and Zimmerman 2010). Behavioral plasticity – evolved in response to naturally variable environments – may allow species to persist in the face of more severe changes arising from human activities (Van Buskirk 2012). Developing a better understanding of the behavioral, physiological, and molecular mechanisms by which aquatic organisms are able to adjust their reproductive biology to natural variation in flow regimes may therefore also inform predictions about the likely biological impact of anthropogenically manipulated flow rates.

Male three-spined sticklebacks (*Gasterosteus aculeatus*) build nests, which serve both as a focal point for courtship and as a receptacle for eggs and developing fry, that typically consist of collected plant debris glued into a depression dug in a sandy substratum (Van Iersel 1953; Wootton 1976). The nesting glue – “spiggin” – is a multimeric glycoprotein, encoded by a multigene family (Jones et al. 2001; Kawasaki et al. 2003; Kawahara and Nishida 2006, 2007), which is synthesized in the male's kidney and stored in the urinary bladder prior to secretion (Jakobsson et al. 1999). Regulation of gene expression in response to environmental variables potentially allows sticklebacks to alter the type and/or quantity of spiggin synthesized by the kidney, providing a molecular physiological basis for observed plasticity in nest construction (Rushbrook et al. 2010). In this study, we examine how experimentally manipulated water flow regimes experienced by nesting male sticklebacks affect the expression of spiggin genes. First we isolated partial sequences of spiggin genes from the river population under investigation and also from a local pond population, to ensure that any genes potentially expressed only under still-water conditions were not missed. We then reared the laboratory-bred progeny of river-caught sticklebacks under controlled flowing or still-water conditions, and used RT-qPCR to examine patterns of spiggin gene expression among both nesting and non-nesting (control) males.

## Materials and Methods

### Animals and husbandry

**Figure 1:**
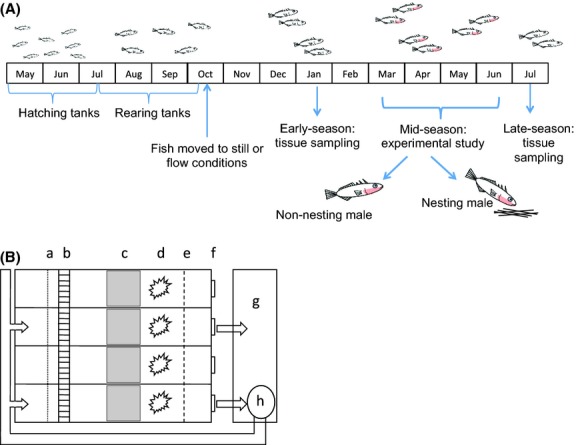
(A) A schematic overview of the experimental design and sampling programme. (B) A diagram of the nesting channels used in the study. Individual channels (45 × 13 × 18 cm) separated by solid plastic barriers were created in large plastic trays (80 × 60 × 20 cm). A 8000-L·h^−1^ water pump moved water from a sump tank via a 32-mm-diameter corrugated hose into two of the four channels in each tray. The water entering the flow channels first passed through a sponge baffle (“a”) and a 50 mm collimator of 5-mm-diameter plastic straws (“b”) to generate a nonturbulent flow of 5 ± 1 cm·sec^−1^ through the nesting area. Water then passed through a mesh barrier (“e”) before exiting the nesting channels via an outflow (“f”). In the remaining two nesting channels in each tank, there was no directional flow, but water quality and circulation was maintained by 50% water exchange every 2 weeks along with air stones and biofilter units. All nesting channels were provided with a (10 × 10 × 1 cm) petri dish of 150 g sand (“c”) and a bundle of 200, 5-cm-long black polyester threads (“d”) as nesting material.

Adult three-spined sticklebacks collected in April 2009 from the River Eye, Leicestershire, UK (52.759°N, −0.814°W), were transported to aquarium facilities at the University of Leicester and maintained at 16 ± 1°C under a 16L:8D photoperiod, to induce sexual maturation. These fish were used as parents for the generation of 13 full-sibling families, generated using standard IVF techniques (Barber and Arnott 2000). Newly-hatched fry were fed infusoria for several days before being switched to a diet of laboratory-hatched *Artemia* sp. nauplii. After 4 weeks, juvenile fish from all families were combined, and 50 randomly selected individuals were transferred to each of six 40 L aquaria, held on a filtered, recirculating system (Fig. 1A). Fish were reared in these aquaria for a further three months before being transferred into their experimental rearing treatments. Throughout the rearing period, fish were kept in conditions designed to track seasonal changes in day length and temperature and fed a mixture of live *Artemia* sp. nauplii and frozen bloodworms (*Chironomus* sp. larvae) supplemented with flake food (Tetra Prima® Spectrum Brands Europe GmbH, Sulzbach, Germany).

### Experimental rearing treatments

At 4 months of age, the juvenile fish in the six rearing aquaria were pooled and distributed evenly between each of six 75-L circular plastic tubs, which provided either the still-water (“still”) or flowing-water (“flow”) treatment. Each tub housed a power filter (Fluval 3 plus, 700 L·h^−1^) in a central column (diameter 10 cm) and was filled to water depth of 30 cm, creating a rearing density of approximately 0.7 fish per liter. In “flow” tubs, a unidirectional water current of 12.0 ± 1.0 cm·sec^−1^ was produced by directing the output of the power filter through a perforated radial spray bar (Barber and Huntingford 1996). In “still” tubs, the filter output flowed directly into the central column, which was allowed to overflow into the surrounding tub; whilst this generated a small amount of turbulent water movement in the tubs, it was nondirectional (0.0 ± 1.0 cm^−2^). Behavioral observations showed that fish in the flow treatment typically swam against the current, often in a polarized school, whereas fish in the still treatment tubs did not behave in this manner. Because the output of the pump was equal in all tubs, filtration rates, temperature, and noise levels were maintained across treatments.

### Nesting study

In mid-January 2010, the water temperature and day length experienced by fish in the rearing tubs were switched to spring conditions (16 ± 1°C, 14L:10D), after which fish were checked each week for signs of sexual maturation. Males developing nuptial coloration (i.e., blue eyes and/or red throats) were then transferred individually to nesting channels (17 ± 1°C, 14L:10D; Fig. 1B). Males reared under the flow treatment were placed in nesting channels with flowing water (5.0 ± 1.0 cm·sec^−1^), and those reared under still conditions were placed in nesting channels with no directional flow. All nesting channels had a water depth of 10 cm, and each contained a square 10 cm petri dish of sand and 200, 5-cm-long black polyester threads as nesting material (Barber et al. 2001).

In the nesting channels, males were presented daily, for 20 min, with a free-swimming, gravid female to stimulate nesting behavior, and were checked daily for signs of nest building. When a male began gluing threads into a nest pit, that is, had reached stage 2 of nest construction (Rushbrook and Barber 2006), the nesting male was removed and dissected (see below). At precisely the same time, a size-matched “control” male – which had experienced the same rearing history and had entered a nesting channel on the same day as the focal nesting male but had not yet started nest building – was also removed and dissected. This approach permitted a paired design, allowing the effects of flow conditions and nest building on gene expression to be quantified whilst controlling for time in the season, body size, and time in treatment.

### Fish dissection and tissue sampling

All male sticklebacks that had been used in the nesting trials (i.e., “mid-season”; Fig. 1A) were then euthanized by benzocaine anesthetic overdose, measured (standard length, to 0.1 mm), and weighed (to 0.001 g). The kidney and liver of each fish were removed and weighed (to 0.0001 g) to calculate indices of sexual development (kidney somatic index (KSI), an indicator of circulating androgen levels in male sticklebacks; Borg and Mayer 1995) and body condition (hepatosomatic index (HSI), which indicates medium term energy reserves; Chellappa et al. 1995). Kidneys were then snap-frozen in liquid nitrogen and transferred to −80°C for storage until RNA extraction (see below).

In addition, to allow an analysis of any effects of flow regime on temporal patterns of body condition or sexual development, a sample of sticklebacks was also dissected 2 days before implementing spring conditions (i.e., “early season”, at ca. 8 months of age, before any fish showed any external signs of sexual maturity; Fig. 1A), as was a sample of males that had failed to exhibit signs of sexual maturation by the end of the nesting trials (i.e., “late season”; Fig. 1A).

### Isolation and sequencing of partial spiggin cDNAs

Total RNA was extracted from a snap-frozen and −80°C-stored kidney of an experimental River Eye male stickleback that had nested under the flowing water regime using an RNeasy Plus Mini kit (Qiagen GmbH, Hilden, Germany) following manufacturer's instructions. Total RNA was also extracted from the kidney of a male stickleback sourced from Brocks Hill pond, a nearby still-water site (52.591°N, −1.088°W), which had nested in a laboratory aquarium under still-water conditions. RNA was eluted into DEPC-treated water and the concentration and purity determined using a NanoDrop spectrophotometer (LabTech International, Lewes, UK). One microgram of total RNA was electrophoresed on a nondenaturing 1.5% (w/v) agarose gel to check for degradation. First strand cDNA was reverse transcribed from one microgram of total RNA using a Quantitect Reverse Transcription Kit (Qiagen GmbH) that incorporates genomic DNA removal prior to reverse transcription. REDTaq ReadyMix (Sigma-Aldrich, Gillingham, UK) was used to amplify partial sequences of spiggin cDNA with two sets of degenerate primers (SPGF1/SPGR1 and SPGF2/SPGR2), based on primers used by Kawahara and Nishida (2006) and designed against a region conserved between all members of the spiggin multigene family (Table 1). The reaction conditions of the PCR were as follows: 94°C for 2 min, followed by 30 cycles of 94°C for 30 sec, 53°C for 30 sec, and 72°C for 1.5 min, with a final extension of 72°C for 10 min. PCR products were eletrophoresed on a 1.5% (w/v) agarose gel, and single bands of the expected size were excised before purifying with a MinElute Gel Extraction Kit (Qiagen GmbH). Purified PCR products were then cloned into pCR®4-TOPO vector using a TOPO TA Cloning for Sequencing Kit (Life Technologies, Carlsbad, CA) following manufacturer's instructions. Clones were isolated from overnight LB cultures using a GenElute Plasmid Miniprep Kit (Sigma-Aldrich) prior to Sanger sequencing by Genome Enterprise Limited (Norwich, UK). Nucleotide sequences of the partial spiggin cDNAs were processed in Geneious Pro 5.6.3 (Drummond et al. 2012) to remove vector before using BLASTN to search the NCBI nonredundant (nr) database for confirmation that the obtained cDNAs were *G. aculeatus* spiggin gene products. To discriminate between spiggin type 1 and type 2 for optimal qPCR primer design, the 3′ ends of these genes were obtained by 3′ RACE using a GeneRacer Kit (Life Technologies) and the gene-specific primers SPG3R01 and SPG3R05 (Kawahara and Nishida 2006). All sequences were submitted to dbEST and given the following accession numbers: dbEST: 77489567-77489625 and GenBank: JK993477–JK993535. These sequences were aligned along with published spiggin genes from GenBank using the Geneious alignment tool with default settings in Geneious Pro 5.6.3.

**Table 1:** PCR primers used in this study.

Primer name	5′–3′ sequence	Purpose in study	Annealing temp
SPGF1	CCAGCATATCTTTAAATACGG	Degenerate PCR	53°C
SPGR1	SATGGAGGACACCCAGTAAAY	Degenerate PCR	53°C
SPGF2	CGAGTTGATCAGAGACAGCAAGC	Degenerate PCR	53°C
SPGR2	GTCACAAACKGGCCYTCAATGAC	Degenerate PCR	53°C
SPG3R01	GATGTGTCATTGAAGGCCAGTTTGT	3′ RACE	65°C
SPG3R05	CTACCAGGAACTCACTGAAAGCTGTG	3′ RACE	65°C
Spg alpha F	TGAAAACCAAGAACTGTCTGCAAG	qPCR	66°C
Spg alpha R3	TTTAGGAATACAGCGATAGCCCTTTT	qPCR	66°C
Spg type 1 F2	AAGAAATCAAGGACTGTGTGCAAT	qPCR	65°C
Spg type 1 R1	ACTGCTGGACCCTTTTCCCTATAT	qPCR	65°C
Spg type 2 F2	AACCAATCCAAGTCCGATGACA	qPCR	60°C
Spg type 2 R3	TCGGAAAGAACCCGGTTTC	qPCR	60°C
Ribo L8 F	CGACCCGTACCGCTTCAAGAA	qPCR	60°C
Ribo L8 R	GGACATTGCCAATGTTCAGCTGA	qPCR	60°C
Ribo L13A F	CACCTTGGTCAACTTGAACAGTG	qPCR	60°C
Ribo L13A R	TCCCTCCGCCCTACGAC	qPCR	60°C
Ubiq F	AGACGGGCATAGCACTTGC	qPCR	60°C
Ubiq R	CAGGACAAGGAAGGCATCC	qPCR	60°C

### Reverse-transcription quantitative PCR (RT-qPCR) analyses

Reverse-transcription quantitative PCR (RT-qPCR) analysis was performed to examine the expression of three spiggin genes (spiggin alpha (*Spg-a*), spiggin type-1 (*Spg-1*), and spiggin type-2 (*Spg-2*); see Results) in kidney tissue of nesting and non-nesting male pairs. Primers were designed to be specific to the spiggin gene of interest (Table 1) using Primer 3 (Untergrasser et al. 2012) to generate PCR products of between 100 and 200 bp. Nonspecific amplification of spiggin genes was checked using *Spg-a*,*Spg-1*, and *Spg-2* cloned plasmid DNA. Total RNA was extracted from snap-frozen (−80°C) kidneys from the experimental sticklebacks using the RNeasy Mini Plus Kit (Qiagen GmbH) following manufacturer's instructions. Concentration and quality of the total RNA was determined as above. First strand cDNA was reverse transcribed from 0.5 *μ*g total RNA as above and diluted 1 in 4. The RT-qPCR mixture consisted of 10 *μ*L SYBR Green JumpStart Taq ReadyMix (Sigma-Aldrich), 250 nM of forward and reverse primers (Table 1), 1 *μ*L diluted cDNA and sterile water in a total volume of 20 *μ*L. The RT-qPCRs were performed in duplicate on a Chromo4 qPCR thermocycler (BioRad Laboratories, Hercules, CA) with the following cycling conditions: 95°C for 3 min, followed by 40 cycles of 95°C for 30 sec, 60–66°C (depending on gene amplified – see Table 1) for 30 sec, and 72°C for 30 sec. A melting curve step (50–95°C) was then performed to ensure that only a single product had been amplified in each reaction. Standard curves were performed for each primer pair on the same plate as the experimental samples with a dilution series of cDNA. For each spiggin gene, cDNA from each of the nesting and non-nesting stickleback pairs in each flow regime was run in duplicate qPCRs. “No template” and “no reverse transcriptase” controls were also performed for each primer pair and cDNA, respectively. To normalize the gene expression data, geNorm software (Vandesompele et al. 2002) was used to select the most stable reference gene from the following candidates used in previous three-spined stickleback studies: ribosomal protein L8 (Geoghegan et al. 2008), ribosomal protein L13A, and ubiquitin (Hibbeler et al. 2008). The gene for ribosomal protein L8 was considered to be the most stable reference gene by geNorm and was used to normalize the data.

### Data analysis

Proportional data (i.e., KSI and HSI) were normalized by arcsine-square-root transformation prior to parametric statistical analysis. Factorial (two-way) ANOVAs were used to test the effects of time in the season (early/mid/late) and rearing treatment (flow/still), and their interaction, on HSI and KSI of all sampled fish, and also to test the effects of rearing treatment and nesting status (control/nester) on the HSI and KSI of mid-season males used in the experimental study. For the analysis of the gene expression data, we first calculated the delta Ct value for each sample by subtracting the average reference Ct value from the average target Ct value. We then used MANOVA on the delta Ct values to determine the effects of the two factors of interest (flow regime and nesting status) on the expression of all three genes. We then undertook factorial (three-way) ANOVA to determine the effects of rearing regime (flow/still), nesting status (nester/control), and gene identity (*Spg-a, Spg-1,*and *Spg-2*) on levels of gene expression.

## Results

### Isolation and sequencing of partial spiggin cDNAs

**Figure 2:**
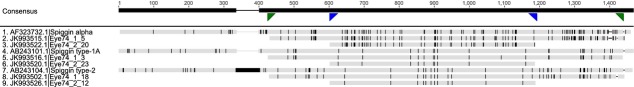
Alignment of spiggin alpha (*Spg-a*), spiggin type-1A (*Spg-1*), and spiggin type-2 (*Spg-2*) with selected partial spiggin cDNAs amplified with SPGF1/SPGR1 (green triangles) and SPGF2/SPGR2 (blue triangles) degenerate primers. Light grey indicates consensus, and black indicates nucleotide differences in each alignment.

The degenerate primers SPGF1/SPGR1 and SPGF2/SPGR2 amplified 1 Kb and 600 bp PCR products, respectively, which were subsequently cloned into pCR®4-TOPO vector and sequenced. Following vector trimming, the DNA sequences of 40 clones from the River Eye fish and 19 clones from the Brocks Hill pond fish were matched to NCBI databases using BLASTN, and aligned with published spiggin genes from GenBank that identified three groups of spiggin gene (E value = 0.0; Similarity = 98–100%; Fig. 2). The first group consisted of spg4 [GenBank: AB221483] and spiggin alpha [GenBank: AF323732] and its alternatively spliced variants, spiggin beta [Genbank: AF323733] and spiggin gamma [GenBank: AF323734]. As spiggin alpha was isolated from a Swedish stickleback population (Jones et al. 2001) and spg4 was isolated from sticklebacks from the Pacific Ocean group (Kawahara and Nishida 2006), the spiggin alpha/spg4 clones will be referred to as spiggin alpha (*Spg-a*) hereafter. The second group consisted of spiggin type-1C [GenBank: AB243103] and the alternatively spliced variants, spiggin type-1B [GenBank: AB243102] and spiggin type-1A [GenBank: AB243101]. As with *Spg-a*, the partial spiggin isolated here was conserved between all variants and so these partial spiggin clones will be referred to as spiggin type-1 (*Spg-1*) genes hereafter. The third group consisted only of spiggin type-2 (*Spg-2*) [GenBank: AB243104].

### Sexual development and body condition of sticklebacks in the study

**Figure 3:**
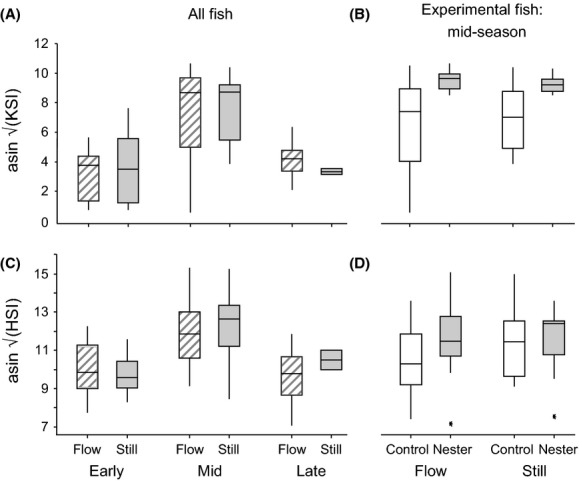
(A) Kidney somatic index (KSI) of flow- and still-reared male sticklebacks in the study, during early, mid-, and late season. Sample sizes: early, flow *n* = 16; early, still *n* = 16; mid, flow *n* = 31; mid, still *n* = 26; late, flow *n* = 9; late, still *n* = 2. (B) KSI of mid-season control (i.e., non-nesting) and nesting male sticklebacks reared under the flow and still treatments. Sample sizes: early, flow *n* = 16; early, still *n* = 16; mid, flow *n* = 31; mid, still *n* = 27; late, flow *n* = 9; late, still *n* = 2. (C) Hepatosomatic index (HSI) of flow- and still- reared male sticklebacks in the study, during early, mid-, and late season. Sample sizes: flow, control *n* = 13; flow, nester *n* = 13; still, control *n* = 11; still, nester *n* = 13. (D) HSI of mid-season control (i.e., non-nesting) and nesting male sticklebacks reared under the flow and still regimes. Sample sizes: flow, control *n* = 13; flow, nester *n* = 13; still, control *n* = 12; still, nester *n* = 13. Arcsine-square-root-transformed data are presented. Horizontal lines in each box indicate the median value; boxes show interquartile range; whiskers show 95% confidence intervals, and asterisks (*) show outlying data points.

There was a significant effect of time in the season on the kidney somatic index (KSI) of male sticklebacks, with mid-season fish (i.e., nesting and non-nesting males used in the experimental trials) having the largest KSI values; however, there was no effect of flow treatment, either as a main effect or in interaction with time in the season (season: *F*_2,100_ = 39.74 *P* < 0.0005; treatment: *F*_1,100_ = 0.00, *P *=* *0.99; season*treatment: *F*_2,100_ = 0.03, *P* = 0.743; Fig. 3A). Among mid-season (experimental) males, those that had started building nests had significantly larger KSI than those that had not started nest building, but again there was no significant effect of flow treatment on KSI, nor was there a significant interaction (nesting status: *F*_1,50_ = 10.57, *P* = 0.002; treatment: *F*_1,50_ = 0.29, *P* = 0.594; nesting status*treatment: *F*_1,50_ = 0.98, *P* = 0.327; Fig. 3B). Hepatosomatic index (HSI) also varied significantly over the season, peaking at mid-season, but was unaffected by flow treatment (season: *F*_2,100_ = 26.53 *P* < 0.0005; treatment: *F*_1,100_ = 0.80, *P* = 0.373; season*treatment: *F*_2,100_ = 0.79, *P* = 0.455; Fig. 3C). Among mid-season (experimental) males, HSI was not affected by flow treatment and did not differ between nest-building and nonbuilding males (nesting status: *F*_1,50_ = 2.52, *P* = 0.119; treatment: *F*_1,50_ = 0.80, *P* = 0.376; nesting status*treatment: *F*_1,50_ = 0.91, *P* = 0.344; Fig. 3D).

### RT-qPCR analysis of spiggin gene expression in fish reared under different flow conditions

**Figure 4:**
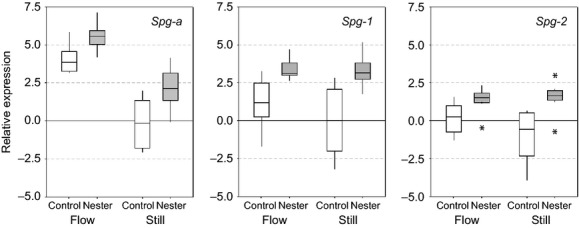
Boxplots showing the effect of flow treatment and nesting status on the expression of three spiggin genes (*Spg-a*, *Spg-1*, *Spg-2*) relative to that of the reference gene, ribosomal protein L8, by male sticklebacks in the study. Horizontal lines in each box indicate the median value; boxes show interquartile range; whiskers show 95% confidence intervals and asterisks (*) show outlying data points. Sample sizes: flow, control *n* = 10; flow, nesting *n* = 13; still, control *n* = 8; still, nesting *n* = 12. See Table 2 for statistical analysis.

Analysis by three-way ANOVA of delta Ct values was undertaken to investigate the role of experimental flow treatment, nesting status, and gene identity on patterns of spiggin gene expression in the kidney tissue of experimental male sticklebacks. This analysis demonstrated that spiggin gene expression was strongly affected by flow treatment, nesting status and gene identity (all *P *<* *0.0005; Table 2). Males reared under the flowing water treatment exhibited higher levels of spiggin gene expression than males reared under still conditions, and nest-building males exhibited higher expression than non-nesting controls (Fig. 4). Furthermore, a highly significant interaction between rearing regime and nesting status was revealed (*P* = 0.006), with high levels of gene expression among non-nesting, flow-reared fish leading to smaller differences in gene expression between nesting and non-nesting fish reared in flowing water than for fish reared in still water. In addition, there was a highly significant interaction between flow regime and gene identity on expression levels (*P *<* *0.005), with expression levels of *Spg-a* being more strongly increased among flow-reared fish than *Spg-1* or *Spg-2*.

**Table 2:** Factorial (three-way) ANOVA investigating the effect of rearing regime, nesting status, and nesting glue gene identity (*Spg-a*, *Spg-1*, and *Spg-2*) on expression level relative to ribosomal protein L8.

Source	df	*F*	*P*
Flow treatment (flow/still)	1	64.39	**<0.0005**
Nesting status (control/nester)	1	111.27	**<0.0005**
Gene identity (*Spg-a*/*Spg-1*/*Spg-2*)	2	43.67	**<0.0005**
Flow treatment * Nesting status	1	7.85	**0.006**
Flow treatment * Gene identity	2	6.12	**<0.0005**
Nesting status * Gene identity	2	1.87	0.159
Flow treatment * Nesting status * Gene identity	2	0.06	0.946
Error	117		
Total	128		

Statistically significant *P* values (at an alpha level of 0.05) are shown in bold.

## Discussion

Our results show that the flow regime experienced by male sticklebacks substantially alters the expression of genes encoding a nesting glue that plays a critical role in stickleback reproductive biology. We used RT-qPCR to quantify the expression of three major spiggin genes in the kidney tissue of nesting and non-nesting (control) males, which had been bred in the laboratory (from river-dwelling parents) and reared under either still- or flowing-water conditions. As expected, expression of all three spiggin genes was significantly higher among nesting than non-nesting males. Our study also revealed a strong effect of flow regime on gene expression, with males reared in flowing water exhibiting higher spiggin gene expression levels than males reared in still water, regardless of their nesting status. Furthermore, both the overall level of expression, and the responsiveness to flow regime, differed between individual spiggin genes. As there was no effect of flow regime on informative indicators of body condition (HSI) or sexual development (KSI) – indicating equivalent energetic and sexual development status of fish under both treatments – we are able to immediately discount the possibility that patterns of gene expression simply reflect an energetic effect of rearing treatment. Taken together, our results demonstrate that sticklebacks adjust spiggin gene expression patterns in response to the flow regimes they experience, and suggest that the molecular and physiological basis of nesting behavior in this species exhibits plasticity in response to environmental cues.

Spiggin acts as an adhesive in nest construction (Wootton 1976; Jakobsson et al. 1999) and so it is not unexpected that nest-building fish show higher levels of spiggin gene expression than non-nesters. However, our results also showed that flow-reared fish increased the expression of spiggin genes – and especially the expression of *Spg-a* – in *advance* of nest building, whereas this was not the case for still-reared fish. Increasing spiggin gene expression prior to construction may serve to “prime” males for nesting under high flow rates, allowing material to be more effectively secured to the substrate and/or as an adaptive response to scarce (or temporally unpredictable) nesting opportunities typically found in rivers (Mori 1995). Under more benign flow regimes, suitable nesting habitat may be more available, and potential nest sites may not require the immediate availability of a large quantity of spiggin for exploitation. As high levels of spiggin gene expression – and subsequent protein synthesis – are costly energetic processes, environmentally induced plasticity in the timing of spiggin upregulation might reflect an adaptive response to the flow-dependent costs and benefits of spiggin expression.

Previous studies have shown plasticity in stickleback nest construction in response to changing flow regimes. In an earlier experimental study, river-caught males were exposed to rapid switches in flow conditions and allowed to construct nests under both flowing- and still-water regimes within 7 days (Rushbrook et al. 2010). In that study, nests built in flowing water were smaller and more elongate than those built in still water, and also contained more spiggin per gram of nest material, suggesting they may have been more tightly secured; however, the total amount of spiggin did not differ between flow- and still-built nests. Our present results suggest that exposure to differential flow regimes over a longer period might be likely to affect the total amount of the spiggin incorporated into nests; however, this could not be tested as males were not permitted to complete nests in this study.

The existence of multiple spiggin genes, (Jones et al. 2001; Kawasaki et al. 2003; Kawahara and Nishida 2006, 2007), suggests a number of gene duplication events during three-spined stickleback evolution. It has been speculated that different spiggin genes, coding for different spiggin protein subunits, might be selected to suit local conditions (Kawahara and Nishida 2007). Our results show that flow regime had different effects on the expression of the three different spiggin genes under investigation. Although expression of all three spiggin genes in this study was significantly increased in flow-reared fish compared to still-reared fish, the increase in *Spg-a* was far greater than that of *Spg-1* and *Spg-2*. The consequences of this larger increase in the component contributed by *Spg-a* for the structure and function of the spiggin protein are as yet unknown.

Our results provide another example of phenotypic plasticity in sticklebacks (see also Candolin 2009; Dingemanse et al. 2012; McCairns and Bernatchez 2012), which may have facilitated their exploitation of a diverse range of environments. Behavioral plasticity potentially allows animals to persist in the face of rapidly changing environments; however, in some cases, plastic behavioral responses that are adaptive under natural levels of variation become maladaptive under more extreme anthropogenic changes (Van Buskirk 2012). The nest-building behavior of three-spined sticklebacks shows population-level variation, with fish inhabiting divergent habitat types building different types of nests (Rowland 1994; Ólafsdóttir et al. 2006; Rushbrook and Barber 2008; Raeymaekers et al. 2009). Our study demonstrates flow-mediated plasticity in the expression of spiggin genes in laboratory-born male sticklebacks descending from river populations. It is possible that this plasticity in gene regulation is an adaptation to life in dynamic, variable flow regimes, but to test this hypothesis, further studies that investigate the level of plasticity across populations that differ in their flow regime would be required.

Fluvial ecosystems are increasingly perturbed by anthropogenic activity, including processes such as dam construction, abstraction, and channel modification (Pringle 2001) and by changes in temporal patterns of rainfall associated with climate change (Döll and Zhang 2010). Such anthropogenic activities can dramatically affect the biology of stream-dwelling organisms; for example, the morphology of the cyprinid fish *Cyprinella venusta* changed rapidly following impoundment of rivers across the Mobile River system in southeastern United States (Haas et al. 2010). Our results suggest that sticklebacks from rivers – which may have evolved strategies to cope with variable water flows – might have the capacity to adjust at both a behavioral and a molecular level to such human-induced alterations to water flow. Whether similar levels of nesting plasticity exist among still-water-adapted populations, and what the fitness consequences of such plasticity would be, remains a fruitful area for future investigation.
